# Post-diapause transcriptomic restarts: insight from a high-latitude copepod

**DOI:** 10.1186/s12864-021-07557-7

**Published:** 2021-06-03

**Authors:** Vittoria Roncalli, Matthew C. Cieslak, Ann M. Castelfranco, Russell R. Hopcroft, Daniel K. Hartline, Petra H. Lenz

**Affiliations:** 1 Pacific Biosciences Research Center, University of Hawai’i at Mānoa, 1993 East-West Rd, Honolulu, HI 96822 USA; 2 Integrative Marine Ecology Department, Stazione Zoologica Anton Dohrn, Naples, Italy; 3 Institute of Marine Science, University of Alaska, Fairbanks, 120 O’Neill, Fairbanks, AK 99775-7220 USA

**Keywords:** Copepod, Gulf of Alaska, *Neocalanus flemingeri*, Transcriptomics, Diapause, Respiration

## Abstract

**Background:**

Diapause is a seasonal dormancy that allows organisms to survive unfavorable conditions and optimizes the timing of reproduction and growth. Emergence from diapause reverses the state of arrested development and metabolic suppression returning the organism to an active state. The physiological mechanisms that regulate the transition from diapause to post-diapause are still unknown. In this study, this transition has been characterized for the sub-arctic calanoid copepod *Neocalanus flemingeri*, a key crustacean zooplankter that supports the highly productive North Pacific fisheries. Transcriptional profiling of females, determined over a two-week time series starting with diapausing females collected from > 400 m depth, characterized the molecular mechanisms that regulate the post-diapause trajectory.

**Results:**

A complex set of transitions in relative gene expression defined the transcriptomic changes from diapause to post-diapause. Despite low temperatures (5–6 °C), the switch from a “diapause” to a “post-diapause” transcriptional profile occurred within 12 h of the termination stimulus. Transcriptional changes signaling the end of diapause were activated within one-hour post collection and included the up-regulation of genes involved in the 20E cascade pathway, the TCA cycle and RNA metabolism in combination with the down-regulation of genes associated with chromatin silencing. By 12 h, females exhibited a post-diapause phenotype characterized by the up-regulation of genes involved in cell division, cell differentiation and multiple developmental processes. By seven days post collection, the reproductive program was fully activated as indicated by up-regulation of genes involved in oogenesis and energy metabolism, processes that were enriched among the differentially expressed genes.

**Conclusions:**

The analysis revealed a finely structured, precisely orchestrated sequence of transcriptional changes that led to rapid changes in the activation of biological processes paving the way to the successful completion of the reproductive program. Our findings lead to new hypotheses related to potentially universal mechanisms that terminate diapause before an organism can resume its developmental program.

**Supplementary Information:**

The online version contains supplementary material available at 10.1186/s12864-021-07557-7.

## Background

Diapause is a type of dormancy that is genetically controlled and widespread among arthropods. It has evolved independently in multiple taxa [1–3]. It is an alternative developmental program that allows organisms to survive during periods of unfavorable environmental conditions and synchronize reproduction and growth to optimize survival [1–3]. The diapause program extends the lifespan of the organism by introducing a period of suspended development [4]. This program has been divided into three major phases: pre-diapause, diapause and post-diapause [4]. During pre-diapause resources are actively accumulated and maturation is postponed. During the diapause phase the organism is in a state of “suspended animation,” characterized by low metabolic rates and increased stress resistance. In post-diapause biological and cellular processes return to an active state and the organism completes its life cycle. While different phases and sub-phases of diapause were recognized early on [2], the physiological progression through the phases remains poorly characterized [4]. The molecular basis of such major physiological transitions is fundamentally interesting, in particular the transition from diapause to post-diapause, which requires the restart of a diverse set of biological processes such as development, metabolic activation, muscle function, cell division and digestion [5, 6]. Here, we investigated this transition in an organism with adult-female diapause, where the primary biological process during post-diapause is the completion of the reproductive program [7, 8].

The sub-arctic calanoid copepod *Neocalanus flemingeri* [9] is a good model for studying emergence from diapause. With one generation per year, diapause is a critical component of *N. flemingeri*’s life cycle. Between March and May, the juvenile stages (copepodids CI to CV) are found in the upper 100 m, where they are actively growing and accumulating lipids in preparation for the diapause phase [10–12]. Starting in May, the population is dominated by pre-adults (copepodid stage CV) that continue to accumulate lipid stores. By June, the pre-adults migrate to depth, molt into the non-feeding adult stage (both males and females) and mate [10, 11]. Adult males die, while the females migrate deeper in the water column (≥ 400 m), and females enter the diapause phase becoming inactive for ca. 5–7 months (from summer until December/January) [10]. As part of the post-diapause phase, females initiate the reproductive program that takes ca. seven weeks [13]. As eggs near maturity, females ascend to shallower depth (250–500 m), start spawning and newly recruited young copepodids (CI) appear in the upper 100 m in March [10, 11].

Stimuli associated with the collection of diapausing females from depth with a plankton net were found to set in motion the transition from diapause to post-diapause in *N. flemingeri* [13]. By one-week post collection the reproductive program had started as revealed by weekly transcriptomic profiling of females between time of collection and end-of-life. Completion of the reproductive program involves sequential activation of genes involved in germline development, meiotic cycle and oogenesis [13–15]. The program is paired with regulation of genes involved in other biological processes associated with metabolism and homeostasis. The up- and down-regulation of genes associated with catabolism (e.g. glycolysis, β-oxidation, proteolysis), homeostasis and cell maintenance suggests shifts in energy source and energy reallocation among processes as oocytes mature and spawning begins [14]. From these studies it was clear that by week one, all females were in the post-diapause phase and the transition from diapause to post-diapause had been missed. Thus, the goal of this study was to understand the transcriptional changes the organism has to go through to complete the transition from the “suspended” diapause state to the fully “active” post-diapause state. Dimensionality-reduction analysis, gene expression profiling and correlation network analysis were applied to identify how and which genes/processes were sequentially regulated as *N. flemingeri* females terminated diapause and entered the post-diapause period.

## Results

### Diapause and post-diapause states have distinct transcriptional phenotypes

**Fig. 1:**
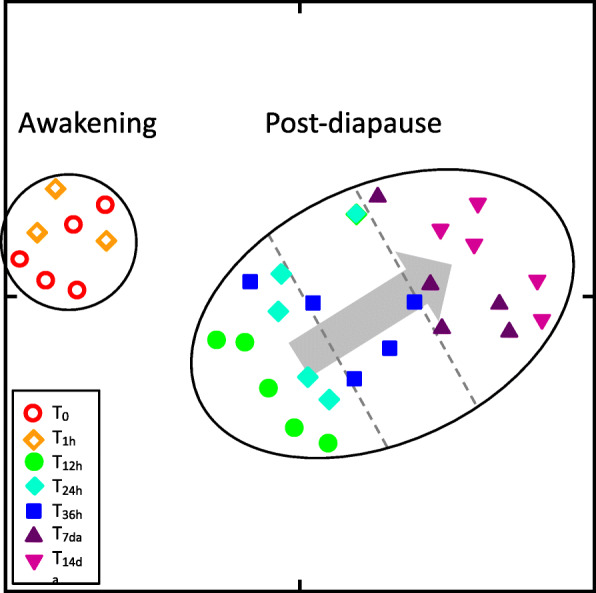
t-SNE analysis of relative expression of all of expressed genes in *N. flemingeri* females from collection (T_0_) to 14 days post-diapause (T_14d_). The analysis includes the log-transformed relative expression (Log_2_(RPKM+ 1)) of all genes (*n* = 140,841) and used perplexity = 9 and number of iterations = 50,000; clusters (enclosed in black ovals) identified using DBSCAN with MinPts = 3 and the Eps value that maximized the Dunn index. Sample timepoints are indicated by different symbols as shown in the inset in the graph. The internal substructure of the post-diapause cluster (indicated by the arrow) highlights the progression from T_12hr_ to T_14d_ samples

The existence of distinct transcriptional phenotypes within a set of samples can be inferred by the approaches described in methods (below) and in particular by the occurrence of clusters in a t-distributed Stochastic Neighbor Embedding (t-SNE) dimensionality-reduction plot. Clustering of the *N. flemingeri* females (*n* = 33) based on the expression of all genes (*n* = 140841), separated them into two distinct clusters; the first cluster included those from the time of collection (T_0_) and those harvested one hour later (T_1hr_), while the second cluster included females from all other samples (T_12hr_ to T_14d_) (Fig. 1). By the 12 h timepoint, the females’ transcriptional phenotype was distinct from the first two timepoints, suggesting that the shift from the “diapause” state to the “post-diapause” transcriptional phenotype had occurred by 12 h post-collection. While 25 females clustered together, a substructure was observed, with samples aligning by timepoint along a diagonal from T_12hr_ to T_14d_ (arrow, Fig. 1). Because t-SNE attempts to retain as much as possible in its 2D representations the proximities found in higher dimensions [16], the non-overlapping pattern of segregated points within this cluster is evidence of progressive changes in gene expression.

Gene expression analysis confirmed the presence of large differences in transcriptional profiles from T_0_ to T_14d_ as indicated by more than 14,000 differentially expressed genes (DEGs) identified across all samples (*p* ≤ 0.05 after FDR correction). Pairwise comparison between T_0_ females and all other timepoints (T_1hr_ - T_14d_) showed a steep up-ramping of the number of DEGs between the 1 hr. and 36 h timepoints (Table 1). Although T_1hr_ females clustered with T_0_ (see Fig. 1) nearly 900 DEGs were identified between these first two timepoints, suggesting that the transition from diapause to post-diapause had already begun at 1 hr (Table 1). Consistent with the clear separation between clusters observed by 12 hr post-collection (see Fig. 1), a 2-fold increase in the number of DEGs was observed comparing T_0_ with T_12hr_ (*n* = 2523) with a similar number of DEGs between T_0_ and T_24hr_ and T_0_ and T_36hr_ (Table 1). The largest number of DEGs was found between T_0_ and T_14d_ (*n* = 4874, Table 1).

**Table 1:** Summary of differential gene expression analysis

	DEGs		
	Total	Up-regulated	Down-regulated
Generalized linear model (GLM)	14608		
Likelihood ratio tests			
T_0_ vs T_1hr_	883	502	381
T_0_ vs T_12hr_	2523	2026	497
T_0_ vs T_24hr_	3346	2524	822
T_0_ vs T_36hr_	3633	2646	987
T_0_ vs T_7d_	2798	1907	891
T_0_ vs T_14d_	4874	2678	2106

Differentially expressed genes (DEGs) were identified using a general linear model (GLM) followed by likelihood ratio tests (FDR; p-value ≤0.05) between T_0_ females and females from T_1hr_, T_12hr_, T_24hr_, T_36hr_, T_7d_ and T_14d_ time points. For each pairwise likelihood test, the number of total DEGs, up- and down-regulated is listed. The pairwise comparisons between other samples are shown in Table S1

### One-hour time point: transient expression of genes associated with diapause termination

The plankton net collection (~ 40 min long net-tow from 400 m depth) generated significant mechanical, chemical and photic stimulation, which induced the termination of diapause. An indication that females were already terminating diapause at T_1hr_ came from the observed down-regulation of genes that are up-regulated during diapause and considered to be biomarkers for the diapause state. Among those, we found pro-longevity genes (Embryonic lethal abnormal vision (ELAV) and Cheerio) and several Phosphoenolpyruvate carboxykinases (PEPCK) genes that are responsible for anaerobic metabolism during dormancy.

**Fig. 2:**
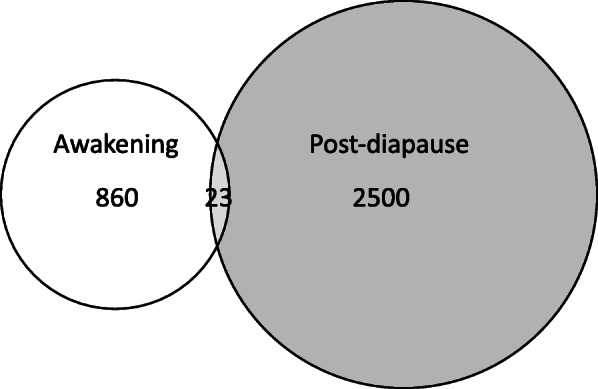
“Awakening” vs “Post-diapause” activation. Venn diagram of the total number of differentially expressed genes (up- and down-regulated) identified in the pairwise comparisons T_0_ vs T_1hr_ and T_0_ vs T_12hr_. DEGs have been identified using the general linear model followed by likelihood ratio tests (FDR; *p*-value ≤0.05) between the indicated timepoints. Only 23 DEGs were shared between the two sets

The T_1hr_ timepoint was also characterized by transient transcriptional changes: 97% of the DEGs were uniquely regulated in 1 hr. females. Only 23 DEGs at T_1hr_ were shared between the two comparisons (T_0_ vs T_1hr_ and T_0_ vs T_12hr_) (Fig. 2). In the comparisons with T_0_, the majority of DEGs were up-regulated (Table 1) at the later timepoints, as would be expected in an organism that is emerging from a state of developmental arrest, low metabolic rates and depressed levels of mRNA.

**Fig. 3:**
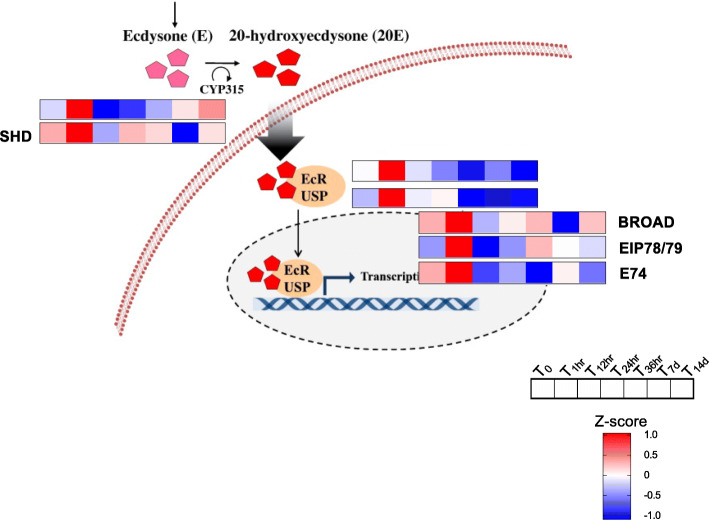
Ecdysteroid signaling pathway in *N. flemingeri* adult females during diapause termination. Schematic representation of the ecdysteroid signaling cascade (adapted from Hwang [17]). Heatmaps show relative expression (z-score) of the *N. flemingeri* genes in females from T_0_ to T_14d_. All genes shown were identified as DEGs

Genes encoding peptides known to be associated with the reactivation of physiological and developmental processes were up-regulated in the T_1hr_ females. At this timepoint, the transient up-regulation was observed for genes involved in the ecdysteroid signaling pathway. This signaling cascade begins with the conversion of the ecdysone (E) to the steroid 20-hydroxyecdysone (20E) mediated by the action of cytochromes P450 (e.g. CYP315, Shade (Shd)). The 20E binds the Ecdysone receptor (EcR) and its dimerization partner Ultraspiracle (USP) triggering the downstream activation of the gene Broad and of the Ecdysone-inducible proteins E74 and Eip78/79. The gene Shd, the receptor genes (EcR, USP) and of the genes Broad, E74 and Eip78/79 were up-regulated at T_1hr_ (Fig. 3). Up-regulation of all genes was transient and only observed at T_1hr_ (Fig. 3).

**Fig. 4:**
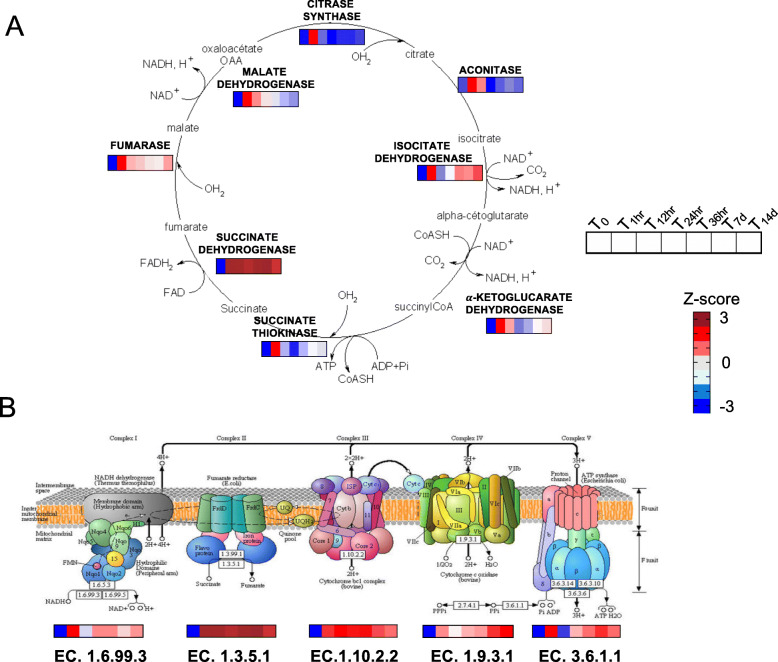
Tricarboxylic acid cycle (TCA) and oxidative phosphorylation. **a** Schematic representation for tricarboxylic acid cycle (TCA) adapted from Wikimedia Commons (https://commons.wikimedia.org/wiki/File:Cycle_de_krebs.png). For each step of the TCA cycle intermediate products, enzymes (bold) and coenzymes (FAD and NAD+) are indicated. For each enzyme, heatmaps show relative expression (z-score)in females from T_0_ to T_14d_. **b** KEGG pathway diagram (map 00190) including gene expression results for the five genes among the DEGs in *N. flemingeri*. The upper part of the figure shows the five respiratory chain complexes with the corresponding E.C. numbers for each enzyme. In the bottom part, heatmaps show relative expression (z-score) of each enzyme associated with the respiratory chain complex in females from T_0_ to T_14d_. All enzymes shown were identified as DEGs. Copyright permission to use and adapt the KEGG map 00190 has been granted from KEGG database [18]

Concurrent with the regulation of genes involved in the 20E pathway, the immediate and transient up-regulation at T_1hr_ included two pathways associated with energy: the tricarboxylic acid (TCA) cycle and the oxidative phosphorylation pathway. The genes encoding the eight enzymes involved in the TCA cycle (Citrate synthase, Aconitase, Isocitrate dehydrogenase, α-ketoglutarate dehydrogenase, Succinate thiokinase, Succinate dehydrogenase, Fumarate, Malate dehydrogenase) were uniquely up-regulated at T_1hr_ (Fig. 4a). Only one gene, encoding the enzyme Succinate dehydrogenase, maintained high expression during the remaining 14 days of the experimental period (Fig. 4a). Genes encoding enzymes involved in the oxidative phosphorylation pathway were also up-regulated at T_1hr_ (Fig. 4b); however, in contrast to the TCA, the high expression of these genes was not exclusive to this timepoint. For NADH dehydrogenase, Cytochrome C oxidase and Nucleosome remodeling factor 38kD (Nurf-38), the relative expression decreased at T_12hr_, but then increased again at T_24hr_ remaining high to T_14d_ (Fig. 4b). Interestingly, genes encoding enzymes involved in glycolysis and β-oxidation were not among the up-regulated DEGs at T_1hr_. These genes did not became significantly up-regulated until later (see below).

In addition to the specific pathways regulated at T_1hr_, we observed reactivation of transcription processes, as suggested by the up-regulation of genes involved in histone acetylation and by the down-regulation of genes associated with chromatin silencing which is one of the processes that prevent RNA metabolism during dormancy. These results are discussed below as part of the WGCNA analysis.

### From collection to 14 days post-diapause – an orchestrated progression of transcriptional changes

**Fig. 5:**
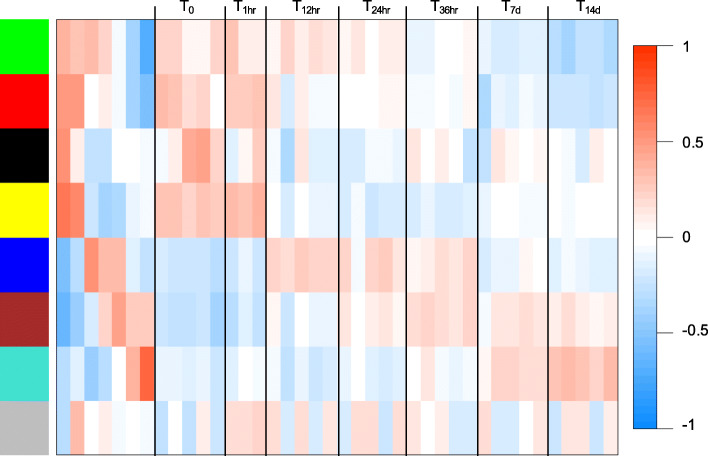
Correlation of WGCNA modules for the DEGs to sample traits. Heatmap shows correlation of module eigengenes (rows labeled by color) to samples either grouped by preservation time point (first seven columns) or individually. The right most columns (*n* = 33) present the correlations of the eigengene expression for each module with the individual samples as labeled on the top. The color of each cell represents the direction and strength of the correlation (blue = negative and red = positive; color scale on right). Number of DEGs in each module: green (*n* = 1409), red (*n* = 1240), black (*n* = 174), yellow (*n* = 1454), blue (*n* = 2909), brown (*n* = 2391) and turquoise (*n* = 3620). The grey module (*n* = 1411) includes DEGs that did not aggregate with a specific gene correlation network

**Fig. 6:**
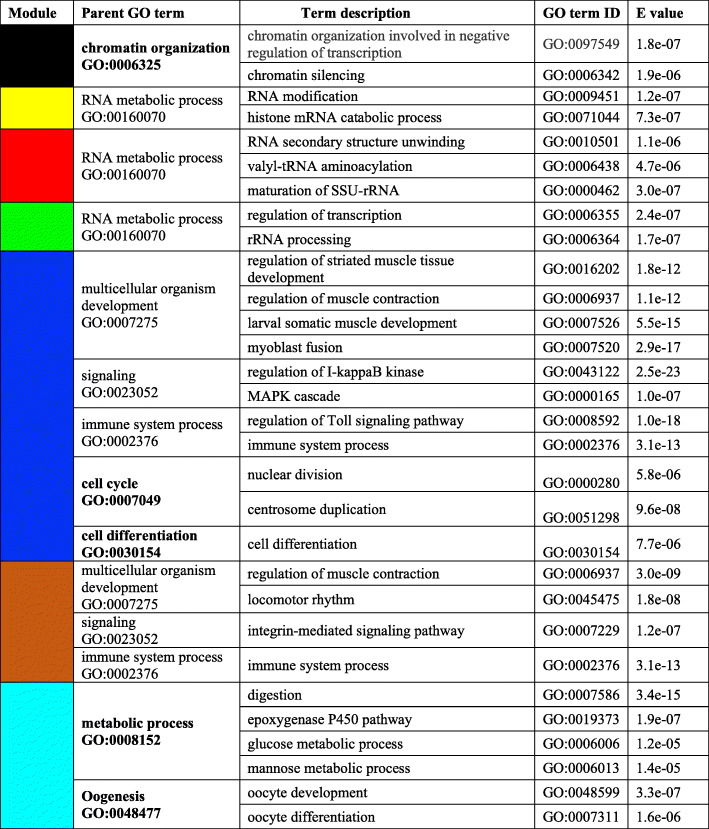
Overrepresented process in females from collection to 14 days post-diapause. List of GO terms enriched for the differentially expressed genes (DEGs) in seven WGCNA modules as shown in the module correlation heatmap (see Fig. 5). Enrichment analysis was performed independently for the DEGs in each WGCNA module against the annotated reference transcriptome (*n*=59544). Module colors refer to Fig. 5. For each enriched GO term, parent GO terms (based on Gene Ontology hierarchical assignment), term description, GO ID and p-value adjusted for FDR are listed. Parent GO terms that were enriched exclusively in one module are in bold

Transcriptional changes during the 14 days were further analyzed using weighted gene correlation network analysis (WGCNA) on the DEGs. Based on expression patterns, the differentially expressed genes across samples (*n* = 14608) were grouped into seven gene network modules that were positively correlated with one or more timepoints (Fig. 5). Six of the seven modules grouped the first two timepoints (T_0_ and T_1hr_) together as would be expected from the t-SNE clusters. However, the black module, with the smallest number of DEGs (*n* = 174), differentiated T_0_ samples from the others. Approximately 10% of the DEGs were placed into the gray module that collects the genes that did not aggregate into a specific gene correlation network (Fig. 5). Enrichment analysis of the DEGs in each module identified overrepresented GO terms belonging to nine biological processes of which five were unique to single modules (Fig. 6).

RNA metabolic process was an enriched process that characterized early changes in gene expression. It was overrepresented for the DEGs identified in two modules that were positively correlated with the T_0_ and T_1hr_ timepoints (red and yellow, Fig. 6). This process was also enriched for the DEGs characterized by a more gradual change in relative expression over the time series (green, Fig. 6). This signal was preceded by a single enriched process (chromatin organization) in the black module that differentiated the T_0_ from all the other timepoints including T_1hr_. These four modules were all positively correlated with early timepoints. In contrast, the blue and brown modules were positively correlated with intermediate and later timepoints starting at T_12hr_ and T_24hr_, respectively (Fig. 5). The blue and brown modules, which together contained more than 5000 DEGs, shared three enriched processes (multicellular organism development, signaling, and immune system process). Two additional enriched GO terms were unique to the blue module (cell cycle, cell differentiation) (Fig. 6). The turquoise module, with the largest number of DEGs (n > 3000), showed positive correlation with the last two timepoints (T_7d_ and T_14d_) and included two enriched processes that were unique to this module (oogenesis, metabolic process) (Fig. 6).

### Early responding biological processes: chromatin organization and RNA metabolism

**Fig. 7:**
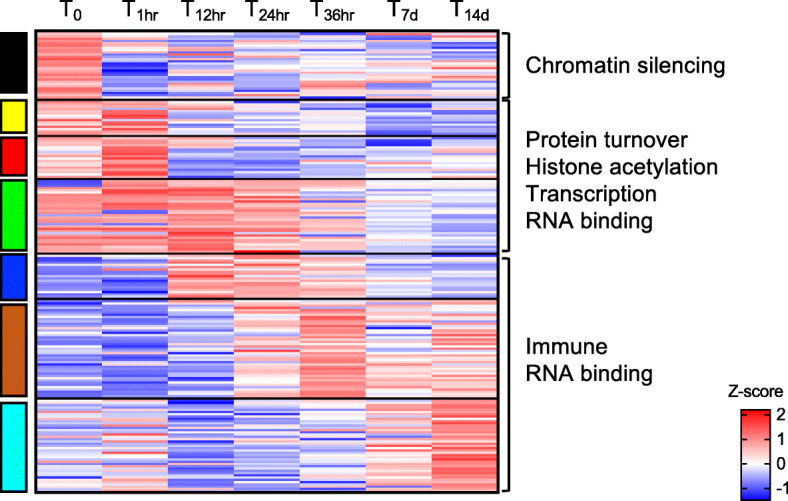
RNA metabolism and chromatin silencing. Heatmap of the differentially expressed genes (*n* = 198) annotated with GO terms associated with chromatin silencing and RNA metabolism (see Fig. 6). Genes (rows) were ordered based on modules (left) for which they were enriched (see Fig. 5). For each gene, relative expression is shown as the average z-score for each timepoint as indicated by the color scale. Timepoints are indicated at the top of the heatmap. Labels on the right indicate processes that were highly represented in each module

Chromatin organization and in particular chromatin silencing were the processes overrepresented for the DEGs within the black module (Figs. 5, 6). Up-regulation of chromatin silencing is not surprising since suppression of transcriptional activities are characteristic of the dormant state. Considering all DEGs (*n* = 198) involved in chromatin organization and RNA metabolism (Fig. 7) 34 were exclusively up-regulated at T_0_ with respect to later timepoints and annotated as Longitudinal lacking proteins (LOLA), Proliferation- associated SNF2, Histone-lysine N-methyltransferase and Lamins. For some LOLA a significant increase in expression was observed again at T_14d_ consistent with an earlier study [14].

DEGs annotated to RNA metabolism, a process that was overrepresented in three modules (red, yellow, green), were characterized by a complex pattern of changes in gene expression suggesting an organized progression of up- and down-regulation of specific genes during the 14 days after collection (Fig. 7). Seven different GO terms were enriched for this process and these include RNA modification, rRNA processing, regulation of transcription and histone mRNA catabolic process (Fig. 6). Several transcription factors (Sp1, Sp4, Pur-alpha), antioxidants (Peroxiredoxin-5) and genes associated with protein turnover (e.g. E3 ubiquitin-protein ligase Siah1, E3 ubiquitin-protein ligase) and histone acetylation (e.g. KAT8 regulatory NSL complex, PolyA-RNA polymerase, Histone demethylation 1) were included in the yellow module, with high expression restricted to the T_0_ and T_1hr_ timepoints. The red and green modules included transcription related genes (e.g. Purine-rich single stranded DNA biniding protein, tRNA pseudouridine) and genes involved in RNA binding (e.g. Eukaryotic translation initiator factor 4, La-related protein CG11505). Although RNA metabolism was not enriched in the other modules (blue, brown, turquoise) some DEGs associated with this process were up-regulated at later timepoints. These DEGs were annotated with additional GO terms indicating that they were also involved in other biological functions such as the immune system (e.g. Embryonic polarity protein dorsal, Toll protein, Spaetzle protein) which is consistent with the enrichment of this biological process in both blue and brown modules (Fig. 6).

### Early post-diapause phase: from 12 to 36 h, overrepresentation of cell cycle and cell differentiation

**Fig. 8:**
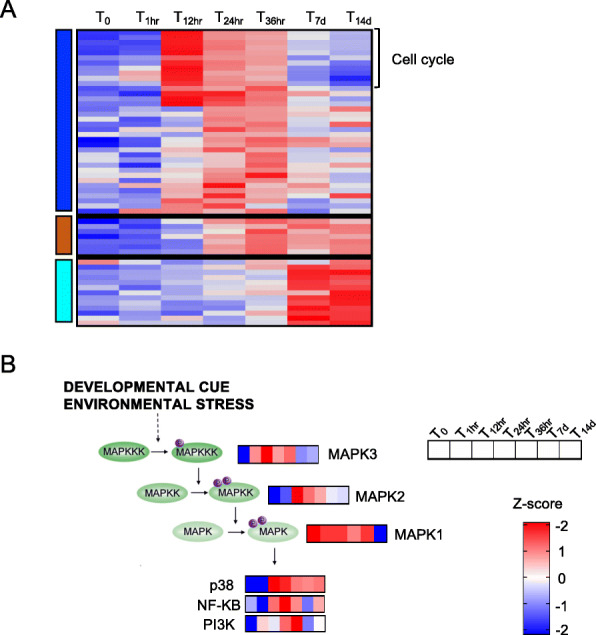
Cell cycle cell differentiation and MAPK cascade pathway. **a** Heatmap of the differentially expressed genes (*n* = 56) annotated with GO terms associated with cell cycle and cell differentiation (see Fig. 6). Genes (rows) were ordered based on modules (left) for which they were enriched (see Fig. 5). For each gene, relative expression is shown as the average z-score for each timepoint as indicated by the color scale. Timepoints are indicated at the top of the heatmap. Labels on the right indicate processes that were highly represented in each module. **b** Schematic representation of the mitogen-activated protein kinases (MAPK) pathway (adapted from Jagodzik et al., [19]. Heatmaps show relative expression (z-score) of the *N. flemingeri* genes in females from T_0_ to T_14d_. All enzymes shown were identified as DEGs

Enrichment of cell cycle and cell differentiation was unique to the DEGs in the blue module, which included genes that were positively correlated with the beginning of the post-diapause period (T_12hr_ to T_36hr_; Figs. 5, 6). Two specific processes within cell cycle were enriched: centrosome duplication and nuclear division (Fig. 6). Consistent with a putative increase in cell proliferation, cell differentiation was also overrepresented during these timepoints (Fig. 6). Among the genes involved in cell cycle, there were several Cyclins and Cyclin-dependent kinases (Cdk) associated with G1/S (CycA, Cdk12, Cdk17, (CycB, CycM2) phases (Fig. 8a). Genes involved in germline development, which also includes ovarian follicle development, were up-regulated between T_24hr_ and T_36hr_ and they included Neurogenic delta, Innexins 2 and Septins-7 (Fig. 8a). The expression of these genes suggests that activation of the oogenesis signal already occurs at T_12hr_. Additional genes involved in cellular differentiation were assigned to the turquoise module and these were most highly expressed in the last two timepoints (T_7d_ and T_14d_). Among these genes, were several cAMP kinases and Crumbs proteins (95F) and some Bicaudal D, Aurora kinases and Innexins 2.

### Post-diapause phase: from 12 h to 14 days, enrichment of signaling processes, multicellular organism development and immune system

Signaling was an overrepresented process for the DEGs within the blue and the brown modules although different GO terms were enriched in each module (Fig. 6). Of particular interest was the gene expression pattern associated with the MAPK cascade and the regulation of I-kappaB kinase/NF-kappaB pathway. These GO terms were enriched for the blue module, which was positively correlated with first stages of post-diapause, T_12hr_ to T_36hr_ timepoints. Activation of the MAPK cascade is essential for many cellular processes, such as cell division and cell differentiation. Furthermore, the MAPK pathway is responsible for the downstream activation of I-kappaB kinase/NF-kappaB pathway. Here, several mitogen-activated protein kinases (MAPK3, MAPK2, MAPK1, p38b) and genes associated with the NF pathway (e.g. PI3K, NF-kappa-B-activating protein) were up-regulated in *N. flemingeri* females between T_12hr_ and T_36hr_ (Fig. 8b**)**. Expression of these genes was significantly lower at T_7d_ and T_14d_ timepoints (Fig. 8b).

**Fig. 9:**
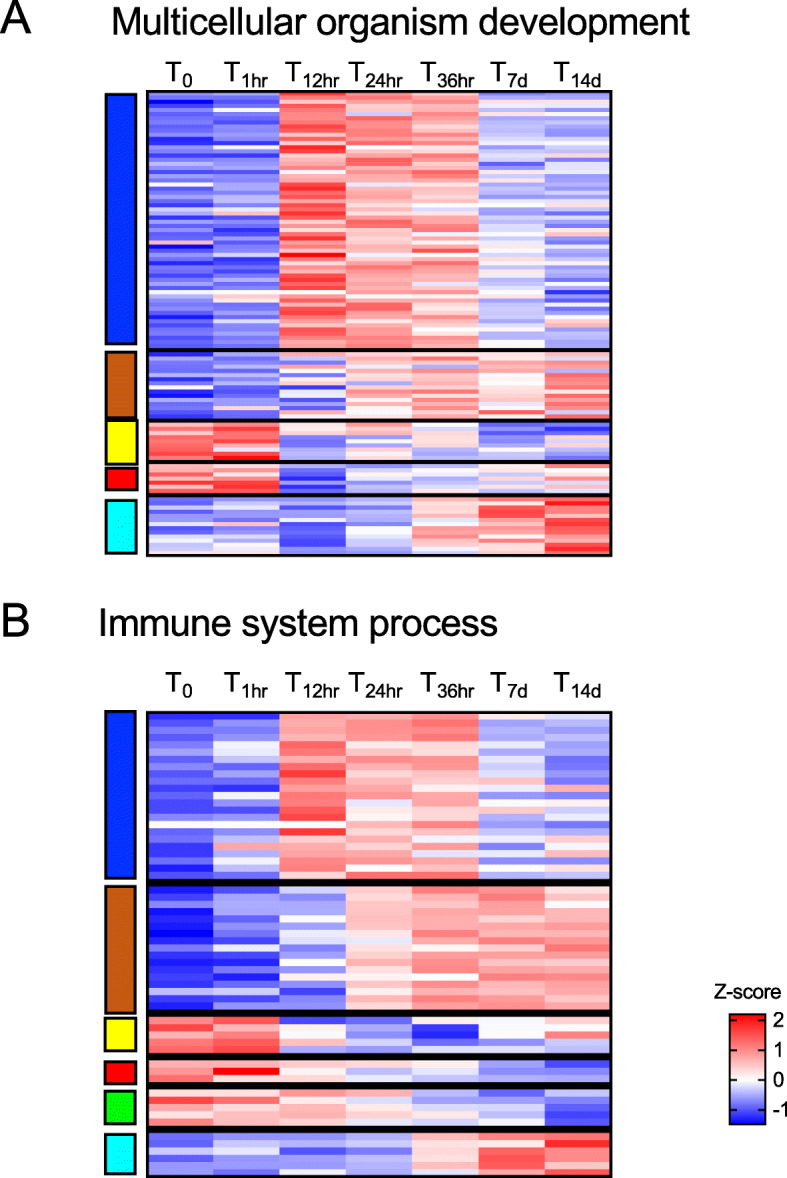
Multicellular organism development and immune system process. Heatmap of the differentially expressed genes annotated with GO terms associated with: **a** multicellular organism development (*n* = 108) and **b** immune system process (*n* = 61) (see Fig. 6). Genes (rows) were ordered based on modules (left) for which they were enriched (see Fig. 5). For each gene, relative expression is shown as the average z-score for each timepoint as indicated by the color scale. Timepoints are indicated at the top of the heatmap

A large group of genes in the blue and brown modules were enriched for GO terms associated with tissue homeostasis such as regulation of muscle contraction (which was shared between the two modules), locomotor rhythm and myoblast fusion (Fig. 6). These GO terms, which shared the same GO parent (multicellular organism development), were positively correlated with T_12hr_ - T_14d_ timepoints (Fig. 9a). Up-regulated genes included Myosins (light chain, muscle), Titin and Troponin T. Even if muscle GO terms were not enriched in other modules, some DEGs associated with this process, annotated as Myosins,Irregular chiasm C-roughest protein abd Muscle LIM proteins, were up-regulated at earlier timepoints (T_0_-T_1hr_).

Another biological process overrepresented for both the blue and brown modules was immune system process, which included Toll signaling pathway as an enriched GO term (Fig. 6). Most of the up-regulated genes were annotated as Toll protein, Speatzle proteins and Embryonic polarity protein dorsal. Relative expression for most of these genes was high in the later timepoints (from T_12hr_ to T_14d_) (Fig. 9b). Some transcripts encoding Spaetzle proteins were also significantly regulated at the earlier timepoints (T_0_-T_1hr_) compared with the other timepoints, which is not surprising considering that regulation of immune system is also required for maintaining tissue homeostasis (Fig. 9b).

### One to two-week post-diapause: overrepresentation of oogenesis and metabolic processes

**Fig. 10:**
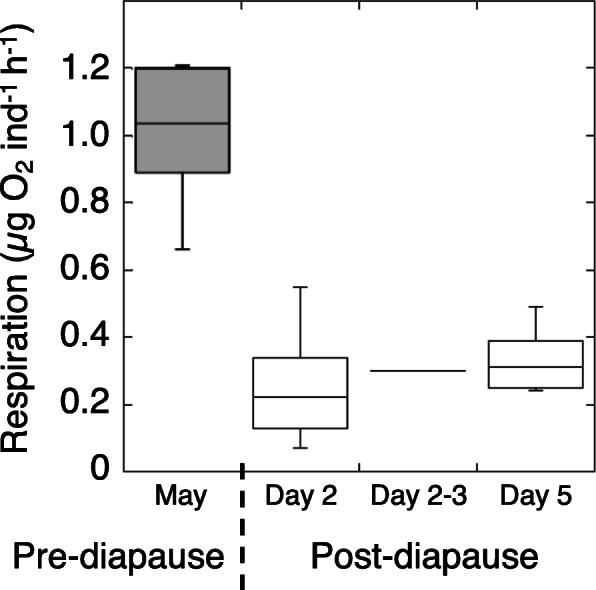
Reproductive program and metabolic processess. Heatmap of the differentially expressed genes annotated with GO terms associated with **a** oogenesis (*n* = 54) and **b** metabolic processes: glycolysis (*n* = 9), *β*-oxidation (*n* = 4), lipase activity (*n* = 27), epoxigenase activity (*n* = 9) and digestion (*n* = 19) (see Fig. 6). Genes (rows) were ordered based on modules (left) for which they were enriched (see Fig. 5). For each gene, relative expression is shown as the average z-score for each timepoint as indicated by the color scale. Timepoints are indicated at the top of the heatmap. Labels on the right indicate processes that were highly represented in each module

By T_7d_ and T_14d_ the reproductive program was in progress as suggested by the enrichment of two GO terms associated with oogenesis (Fig. 6). Of the 54 DEGs annotated to those GO terms, 84% had transient up-regulation at T_7d_ and T_14d_ (turquoise module) (Fig. 10a). The majority of these genes were involved in processes occurring during the early phase of oogenesis such as maintenance and division of germ-line stem cells (e.g. Armadillo segment polarity, Dicer protein, Scrawny protein), oocyte determination and formation of the anterior-posterior axis (e.g. Ovo protein, Egghead protein). The remaining DEGs were grouped into the blue and the brown modules with high expression at the earlier timepoints (Fig. 10a). The blue module included four DEGs annotated as Crumbs protein that are involved in cell differentiation. The brown module (high expression from T_36hr_) included DEGs annotated as Innexin 2, Cueball protein, Brain tumor protein and Maternal protein staufen. These genes are associated with early processes such as ovarian follicle development and oocyte localization that occur at the beginning of oogenesis; this suggests that the reproductive machinery was set in motion starting at T_36hr_ but became overrepresented only at T_7d_ and T_14d_.

The turquoise module was characterized by an overrepresentation of DEGs associated with specific metabolic processes (Fig. 6). Among the enriched GO terms, two terms were associated with carbohydrate metabolism, one with lipid metabolism and one with digestion (Fig. 6). High expression of all nine genes encoding enzymes involved in the glycolytic pathway was found at T_14d_ compared with all other timepoints (Fig. 10b). Six of the nine genes (Hexokinase, Phosphofructokinase, Glyceraldehyde3-phosphate dehydrogenase, Phosphoglycerate kinase, Enolase and Pyruvate kinase) were also up-regulated at T_7d_ (Fig. 10b). A similar pattern was observed in the expression of genes involved in lipid catabolism. Relative expression of all genes involved in β-oxidation as well as several lipases (e.g. Monoacylglycerol lipase, Phospholipase) was significantly higher in T_7d_ and T_14d_ compared with all other timepoints (Fig. 10b). Consistent with the enrichment result, up-regulation of several Cytochromes P450 J, involved in epoxigenase activity, and multiple digestive enzymes (Trypsins) was also observed (Fig. 10b). Eleven out of the 20 Trypsins were even more highly expressed in T_14d_ than in T_7d_ (Fig. 10b). Although Trypsins are usually associated with food digestion, in decapods alteration of their enzymatic activity has been reported during post-larvae development [20]. In their entirety, the gene expression pattern suggests that by the end of the experimental period, the females were entering a period of higher metabolic demands.

### Respiration rates in pre-diapause and early post-diapause females

**Fig. 11:**
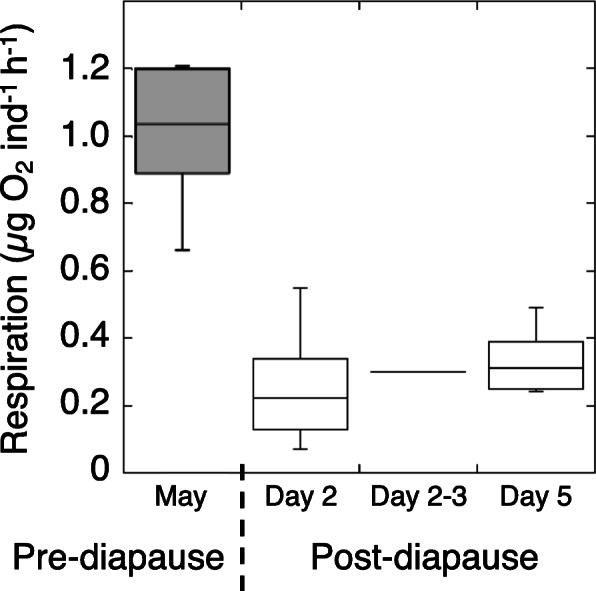
Respiration rate in pre- and post-diapausing *N. flemingeri* females. Summary of respiration rates measured for pre-diapausing “active” (May) and post-diapausing (September) *N. flemingeri* females in a boxplot graph. The box displays the median and interquartile range, while the whiskers give the minimum and maximum values for each time point. Median and mean respiration rates were the same, except for the first post-diapause respiration measurements (median = 0.22, mean = 0.26 μg O_2_ ind^− 1^ h^− 1^)

Differences in respiration rates were recorded between active pre-diapause and post- diapause females. Active *N. flemingeri* females had respiration rates that averaged 1 μg O_2_ per individual per hour (range: 0.66 to 1.2, *n* = 6; Fig. 11). In contrast, respiration rates in females collected from depth and incubated in the laboratory averaged 0.3 μg O_2_ per individual per hour (range: 0.1 to 0.55, *n* = 14) and was similar for all three timepoints post-collection which included days 2, 2–3 and 5 post-collection (Fig. 11).

## Discussion

Diapause, a genetically-controlled suspension of growth and development, can be divided into three main phases: pre-diapause, diapause and post-diapause [4]. The post-diapause phase starts with the reactivation of biological and cellular processes after a period of arrested development and low metabolic rate. Thus, the organism must transition from a state of “suspended animation” to fully active. The post-diapause state varies depending on the next step in the life cycle: the brine shrimp (*Artemia franciscana*) completes embryogenesis; fly pupae (*Sarcophaga crassipalpis*, *Rhagoletis pomonella*) metamorphose into adults; pre-adult stages of some organisms (e.g. the copepod *Calanus finmarchicus*) complete development by molting into adults; other organisms diapause in the adult stage and complete the reproductive program during post-diapause (mosquito *Aedes aegyptii*, copepod *Neocalanus flemingeri*). From a molecular point of view, post-diapause is characterized by large changes in transcription. Within 1 h of a diapause-terminating stimulus, expression levels of genes encoding heat shock proteins and enzymes involved in signaling and respiration pathways are altered in some organisms [21–24]. However, these studies give an incomplete picture, since changes in gene expression were measured in a small number of genes (< 10), providing limited insights into the extensive changes in state that occur during the transition from the diapause to the post-diapause phenotype. The transcriptomic analysis here revealed that the transition from diapause to post-diapause in *N. flemingeri* females involves the finely-orchestrated sequential regulation of thousands of genes.

Transcriptional profiling supports the existence of a diapause and a post-diapause phenotype in the copepod. Based on the relative expression of all genes (*n* = 140841), females separated into two distinct transcriptional phenotypes with the post-diapause phenotype appearing within 12 h after the termination trigger, a combination of photic, chemical, thermal, pressure and mechanical stimuli during net collection. However, in addition to the two distinct transcriptional states, the t-SNE plot (Fig. 1), designed to preserve local relationships, showed sub-structure within the clusters which is indicative of progressive changes in gene expression over time. Differential gene expression and functional analysis confirmed this: the termination trigger initiated a complex program of gene expression regulation that included transient changes in transcription starting with nearly 900 DEGs between the first two timepoints taken 1 h apart (T_0_ and T_1hr_).

### One-hour timepoint: up-regulation of genes within the 20E-cascade pathway

Before the females can switch from diapause to the post-diapause phenotype, they have to “break” dormancy by regulating a specific set of genes. The one-hour timepoint was characterized by the transient up-regulation of genes involved in the 20E-cascade pathway, a pathway which, in insects, is typically silenced (down-regulated) during dormancy to suppress larval growth [25]. In insects, the 20E transcription cascade promotes direct development and is linked to molting and/or metamorphosis [26], although it may also play a role in diapause termination [27]. Up-regulation of the ecdysone receptor was reported as a double peak in the pupae of the flesh fly *S. crassipalpis* at 1 h and 9 h after the diapause-termination trigger [28]. This may reflect activation of two different processes, the first one linked to the diapause-to-post-diapause transition, the second to the initiation of metamorphosis. The transient up-regulation of these genes in *N. flemingeri* seen only at 1 h post-collection is consistent with a specific role in signaling diapause termination (Fig. 3).

### One-hour timepoint: resumption of metabolism

Another transient signal at 1 h was the up-regulation of genes encoding enzymes involved in two major energetic pathways: oxidative phosphorylation and the tricarboxylic acid (TCA) cycle (Fig. 4). In arthropods, metabolic depression is ubiquitous during diapause. This repression can be achieved in different ways: blockage in the delivery of carbon fuel to the mitochondrion (e.g. *A. franciscana*), repression in the expression of genes associated with energetic pathways (e.g. mitochondrial enzymes involved in oxidative phosphorylation), or covalent modification of existing proteins [5]. In diapausing embryos of *A. franciscana*, which are in a deep state of arrest, inhibition of the enzymatic steps within mitochondrial activity occurs during entry into diapause [5]. Hydration-induced termination of diapause in gastrula-stage embryos triggers a transient up-regulation in the expression of two mitochondrial genes (COXI and COXII) in *A. franciscana* [24]. In *N. flemingeri*, genes associated with all five complexes in the oxidative chain were up-regulated at 1 h. In the T_1hr_ females, the peak was transient for genes associated with complexes I, IV and V. Expression of these genes was significantly lower at 12 h, although levels remained above T_0_. For the other two complexes (II and III) gene expression remained high for the remainder of the study (14 days).

An increase in metabolic rate between the diapause and post-diapause phases is universal in arthropods [6, 29]. In T_0_ *N. flemingeri* females*,* expression levels of the eight transcripts encoding TCA enzymes are very low in comparison with the post-diapause levels reported in females starting early oogenesis but prior to spawning [14]. Surprisingly, we found that the increase in expression of these genes occurred before the females had transitioned from the diapause to the post-diapause phase based on t-SNE clustering. At 1 h, we observed a sharp, high peak in expression for all eight transcripts encoding TCA enzymes; at least 3-fold increases in expression were found between T_0_ and T_1hr_ followed by a decrease in expression at 12 h in seven of the eight transcripts. The initial up-regulation of the TCA genes at T_1hr_ preceded any changes in the expression of genes involved in other energetic pathways such as glycolysis and β-oxidation that were observed at T_7d_, consistent with earlier observations [14].

### Respiration rates remain low into post-diapause

Respiration rates are depressed during diapause in all organisms in which they have been measured. However, return to basal levels may not occur immediately, instead, increase of respiration rates can be biphasic as shown in insect pupae during post-diapause [29]. Respiration rates in *N. flemingeri* females averaged about 25% of fully active rates during the first 4 days post-collection (Fig. 11). This is similar to what has been reported for *C. finmarchicus* collected from depth [30, 31]. While these studies suggested that the 75% reduction represented respiration rates during diapause, our transcriptomic data indicated that in *N. flemingeri* the lowered values extend to post-diapause rates, after the up-regulation of genes involved in the TCA cycle and oxidative phosphorylation. Thus, respiration rates in copepods during diapause would be expected to be much lower, possibly approaching the 90% depression reported for diapausing pupae [29]. In addition, it is unclear whether *N. flemingeri* post-diapause respiration rates ever return to the high pre-diapause levels, or whether post-diapause respiration rates are biphasic, as reported for insect pupae.

### Post diapause: release from transcriptional repression

Maintenance of low RNA metabolism as well as low protein turnover and low RNA:DNA ratio define the dormant state in diapausing copepods [7, 30, 31]. Not surprisingly, termination of diapause in *N. flemingeri* females included many DEGs involved in RNA metabolism, including several enriched GO child terms in three WGCNA modules (yellow, red and green). Resumption of transcription appeared to occur through two mechanisms: 1) down-regulation of repressors; and 2) up-regulation of genes involved in processes associated with transcription and protein turnover. Chromatin silencing is used in eukaryotes as one mechanism that counteracts transcriptional activity of RNA polymerases to control specific gene expression [32]. *N. flemingeri* is released from a state of transcriptional repression by an immediate down-regulation of genes associated with chromatin silencing (Fig. 7). The expression of several Longitudinal lacking proteins and Laminins, highly expressed in T_0_ females, were down-regulated by 1 h. Expression remained low for the remainder of the experiment of this study and on through mid-oogenesis [14].

In addition to chromatin silencing, release from transcriptional repression may be occurring through the down-regulation of a group of 21 “diapause specific genes”, mostly annotated as Nuclear receptors, Retinoic acid receptors and the Fork head protein P. These are highly expressed during diapause preparation in *C. finmarchicus* pre-adults (CV) (Lenz et al. [33]) as well as in *N. flemingeri* T_0_ females. In the latter, high expression during diapause is followed by immediate down-regulation at T_1hr_. While we know little about the specific function of these genes in copepods, they are likely to be involved in keeping the organism in a state of transcriptional repression. The second mechanism involves restarting transcription and translation, which in the copepod was characterized by up-regulation of genes associated with protein turnover, histone acetylation and RNA binding. The regulation of these genes was immediate (T_1hr_) and complex, characterized by the sequential and sustained up-regulation of multiple genes during the 14-day experimental period.

### Cell cycle is reactivated early during post-diapause

Cell division is arrested during insect diapause [1, 33] and a significant increase in expression of Cyclins B (CycB) has been identified as signaling diapause termination. In the maggot *R. pomonella*, this signal occurred within 24 h of the initial ramp up in metabolic rate during post-diapause [6]. In *N. flemingeri* females, up-regulation of CycB genes had been observed at the one-week timepoint by Roncalli et al. [13]. In the current study, we found that cell cycle genes are already up-regulated in post-diapause T_12hr_ females, suggesting that this process may be one of the first to be activated (Fig. 8). Enrichment of two child GO terms associated with cell cycle was found for the blue module, which included six Cyclins A (CycA) genes with peak expression during early post-diapause in females from the T_12hr_ to T_36hr_ timepoints. By the 7 and 14-day timepoints, relative expression levels had decreased back to levels observed in the diapause phenotype (T_0_ and T_1hr_ females). In *Drosophila melanogaster*, both CycA and CycB and proteins are highly abundant during the G2 phase of the cell cycle. During meiosis I, CycA peaks first during prophase I, while CycB reaches its maximum expression during metaphase I [34]. The sequential change in gene expression observed in *N. flemingeri* suggests that cell division is synchronized and coordinated, and activated soon after the termination of diapause. This is consistent with histological observations of the S-phase of the cell cycle commencing in the ovary of *N. flemingeri* females within 24 h of collection, and in some females in as little as six to 12 h [35].

### Cell growth and differentiation, the next processes activated during the post-diapause sequence

The transcriptional signal suggests that activation of cell growth and differentiation follows soon after the up-regulation of genes involved in cell cycle. In *N. flemingeri* overrepresentation of genes involved in cell differentiation had been reported to coincide with the enrichment of oogenesis GO terms [13]. Several transcripts encoding for Bicaudal D, Neurogenic delta, Innexins 2 and Aurora kinase showed a 2-fold expression increase at 1 week from collection compared with T_0_ females [13]. In the current study, differential expression of genes involved in cell differentiation started even earlier at T_12hr_ and was characterized by the sequential regulation of different sets of genes (Fig. 8). Some of the genes, up-regulated in females from T_12hr_ to T_36hr_ timepoints were annotated as Septins-7, Neurogenic Delta and Innexin 2. As reported earlier, Bicaudal D and Aurora kinases were only up-regulated in T_7d_ and T_14d_ females, with low expression at the earlier timepoints (this study) and at 3 weeks as reported previously [13].

### From post-diapause to reproductive program

In *N. flemingeri,* females mature and mate in the late spring and summer, prior to entering diapause. Upon emergence during the post-diapause phase, they complete their lifecycle and spawn multiple clutches of eggs during the winter [10]. A previous study focused on post-diapause, reported the females’ progression through the different stages of oogenesis from one-week post-collection to just prior to spawning, which starts at 7 weeks after diapause termination [13, 14]. Transcriptional profiling found that the reproductive program involves the sequential regulation of genes involved in multiple processes, such as cell differentiation, meiotic cell cycle, oogenesis and oocyte localization [13]. The current study, focused on the transition from diapause to post-diapause, included two time points, T_7d_ and T_14d_ that overlapped with the earlier study. At those timepoints, we found enrichment of oocyte differentiation, a process that characterizes the early phases of oogenesis (Fig. 10). Approximately 80% of the up-regulated DEGs involved in those enriched processes were specific to T_7d_ and T_14d_ females; many of these genes had been also reported in the earlier study, as differentially expressed between diapausing females and females from 1 to 3-weeks post-collection. The expression of those DEGs returned to lower levels by three to 4 weeks post-collection, confirming their role in the early oogenesis phase [13]. However, it is also clear that the earlier study failed to capture the initial activation of the reproductive program in the post-diapause phase. Prior to the T_7d_ time point, several genes involved in germline processes and cell differentiation, were up-regulated early in post-diapause, starting at the T_24hr_ to T_36hr_ timepoints. Genes like Armadillo segment polarity, Slowmo protein and Dicer peaked at T_24hr_ and T_36hr_ with a return to low expression at T_7d_ and T_14d_ at similar levels to T_0_. Thus, in this study, while the genes annotated to oogenesis may not have been enriched among the DEGs until T_7d_, the reproductive program was activated almost immediately after diapause termination, a conclusion that is consistent with histological observations.

### Activation of maintenance processes during post-diapause

Post-diapause includes a reactivation of basal organismal processes associated with cellular maintenance and homeostasis. In *N. flemingeri*, investment in cellular maintenance during early to mid-oogenesis phase is suggested by the up-regulation and enrichment of DEGs involved in intracellular signal transduction, muscle activity and immune response [13, 14]. Many of these genes were not up-regulated until the two-week timepoint [13, 14], which was confirmed in the present study. However, the earlier time points indicate that some genes involved in cellular maintenance became up-regulated early in post-diapause (Fig. 9). Several GO terms associated with multicellular organismal development and immune system were overrepresented in two WGCNA modules (blue, brown), that included genes positively correlated with females from T_12hr_ to T_14d_. Noteworthy was the transient up-regulation of several Myosins, Titins and genes associated with Toll and MAPK signaling pathways, which were only highly expressed from T_12hr_ to T_36hr_ (Fig. 9).

## Conclusions

Among arthropods, post-diapause in *N. flemingeri* is relatively simple in terms of development, physiology and behavior. During post-diapause, females complete the reproductive program while maximizing survival to assure spawning of mature and fertilized eggs. Other biological processes are limited: females are not growing nor molting, they are developmentally mature, they are neither searching for a mate nor engaging in mating behavior, nor are they feeding. Females are nearly neutrally buoyant and swimming activity is very limited, although they are capable of escape responses to a mechanical stimulus. Nevertheless, the transcriptomic program that ends diapause and transitions to post-diapause is highly orchestrated starting within an hour of an effective termination stimulus.

How the diapause program of lipid-rich copepods will be affected by global climate change remains a major question. Changes in phenology and geographic distribution are expected, but heretofore predictions have not incorporated a physiological understanding of the diapause program. Studies have been hampered by the copepods’ remote habitat and the inability to duplicate their life cycle in the laboratory. The transcriptional profiling of *N. flemingeri* females during diapause has generated a framework for further experimentation and testing of hypotheses related to female reproductive success.

## Methods

### Field collection and laboratory incubation

*Neocalanus flemingeri* adult females were collected from depth on September 21, 2017 in Prince William Sound (near station “KIP2”; depth:~ 589 m; Latitude 60° 27.13′N; Longitude 147° 99.13′W) at night during the fall cruise of the Seward Line Long-term Observation Program (LTOP) (http://www.sfos.uaf.edu/sewardline/). Zooplankton were collected from 400 to 550 m depth with a multiple opening and closing plankton net (0.25 m^2^ cross-sectional area; 150 μm mesh nets; Multinet-Midi, Hydro-Bios) towed vertically. Collections were immediately diluted, and *N. flemingeri* females were live sorted with five individuals preserved individually in RNAlater Stabilization Reagent QIAGEN within 15 min of the retrieval of the net (T_0_). At collection females were vertically oriented, head up with folded firts antennae (A1) and pereopods remoted. They were unresponsive to mechanical stimulation; however, after ca. 1 h, many females had their A1s deployed. Additional undamaged females were sorted and transferred into 2 L jars containing seawater collected from below 400 m and covered in aluminum foil and incubated at 5 °C (~the temperature of collection depth). After checking for survival, females (*n* = 5) were preserved at each of the following timepoints: T_1hr_ (*n* = 3), T_12hr_, T_24hr_, T_36hr_, T_7d_ (one week) and T_14d_ (2 weeks) for RNA-Seq (Table S1). All samples were stored at − 80 °C until further processing.

### RNA extraction, RNA-Seq, expression profiling and clustering analysis

Total RNA was extracted from individual females using QIAGEN RNeasy Plus Mini Kit (catalog # 74134) in combination with a Qiashredder column (catalog # 79654) and shipped on dry ice to the Georgia Genomics Bioinformatics Core (https://dna.uga.edu). Double-stranded cDNA libraries (NGS Stranded) were multiplexed and sequenced using Illumina Next-Seq 500 instrument (High Output Flow Cell, 75 bp, paired end). From each library, reads < 50 bp long and with an average Phred score of < 30 were removed prior to downstream analysis (FASTX Toolkit v.2.0.0; Illumina Basespace Labs). For each individual, expression level was quantified by mapping each quality-filtered library (*n* = 33) against an existing *N. flemingeri* female reference transcriptome (NCBI BioProject: PRJNA32445; GFUD00000000) [36] using bowtie2 software (default settings; v.2.1.0) [37] and kallisto software (default settings; v.0.43.1) [38]. Kallisto software is expressly designed to reduce potential errors associated with ambiguous mapping, thus it is a better fit for gene expression analysis [37, 38]. Hence, counts generated by kallisto were used for the identification of differentially expressed genes. For the counts generated by the bowtie2 mapping, relative expression of the mapped reads was normalized using the RPKM method (reads per kilobase of transcript length per million mapped reads) [39]. This was followed by log_2_ transformation of the relative expression data after adding a pseudocount of 1 to the RPKM value for each transcript (Log_2_ [RPKM+1]). These log-transformed relative expression data were used for the dimensionality-reduction analysis (see below) and to calculate z-scores for each transcript and sample.

The dimensionality reduction method, t-distributed Stochastic Neighbor Embedding (t-SNE) [40] was applied to the normalized expression data (Log_2_ [RPKM+1]for all 33 samples. This clustering and visualization tool, which emphasizes local similarities among transcriptional profiles, is widely used in transcriptomics. The t-SNE as implemented in the R package Rtsne (Rtsne URL: https://github.com/jkrijthe/Rtsne) [41] was used to analyze the entire set of transcripts (*n* = 140841). After testing several values for the controlling parameters, as is standard practice [16, 40], a perplexity parameter of 9 and a maximum number of iterations of 50000 were settled on, and the algorithm was run multiple times to ensure that the output was representative [16]. To provide an objective method of identifying clusters, the density-based clustering algorithm, DBSCAN (with *MinPts* = 3) was applied to the coordinates of the two dimensional t-SNE representation of the samples [16, 42]. The clustering cut-off (*Eps* parameter) was chosen to maximize the Dunn index score [43]. Both the DBSCAN algorithm and the Dunn index were run in R (dbscan: https://CRAN.R-project.org/package=dbscan) [44](clusterCrit: https://CRAN.R-project.org/package=clusterCrit) [45]. In addition, two other widely used dimensionality reduction algorithms were tested on the dataset and the results (Supplementary Fig. 1).

### Differential gene expression and weighted gene correlation network analysis (WGCNA)

EdgeR [46] was used to assess differential gene expression across samples, based on the counts generated from Kallisto mapping (see above). Transcripts with low expression levels (< 1 count per million (1 cpm)) were removed from each sample. As implemented by EdgeR, libraries (*n* = 33) were normalized by a TMM method (trimmed means of M values). Differentially expressed genes across samples were identified using the negative binomial generalized linear model (GLM) using the glmFit function with adjusted p-value for false discovery rate (Benjamini–Hochberg procedure) (FDR; p-value ≤0.05). To identify differences between each timepoint, pairwise comparisons were performed using likelihood ratio tests (glmLRT) (*p*-value ≤0.05, corrected for FDR). A Venn diagram of the DEGs from the pairwise comparisons T_0_ vs T_1hr_ and T_0_ vs T_12hr_ was generated using the R package Eulerr [47]*.*

Patterns of differential gene expression among samples were further explored using weighted gene correlation network analysis (WGCNA) [48]. The WGCNA analysis was performed on the log-transformed (Log2 [RPKM+1]) gene expression of all DEGs identified by the GLM (*n* = 14608). The WGCNA analysis used a signed network type (networkType = “signed”) with a soft threshold power of 6 and minimum module size of 100 transcripts. A signed network type means the construction of the network was based on the strength of positive correlations among the DEGs and negative correlations were ignored.

### Functional analysis

Functional analysis of the DEGs was obtained by searching the *N. flemingeri* reference transcriptome that had been annotated (E-value cut-off =1 e-05) against SwissProt (*n* = 62126) and Gene ontology (GO) databases (*n* = 59544) [36]. To identify overrepresented biological processes, a gene ontology (GO) enrichment analysis was performed on the DEGs grouped in each WGCNA module (network). Briefly, DEGs from each module with GO annotation (> 50% per module) were compared against the annotated transcripts in the reference transcriptome (*n* = 59544) using TopGO software [49] which implements a Fisher exact test with a Benjamini-Hochberg correction (p-value ≤0.05 v. 2.88.0, default algorithm weight01). Heatmaps of the DEGs annotated within enriched GO terms (see Fig. 6) were generated using relative expression as z-scores of the average of the samples for each timepoint.

### Respirometry

Respiration rates were measured for *N. flemingeri* “pre-diapausing“(May) and “post-diapausing” (September) adult females. In May, “pre-diapausing” females were recently molted individuals from pre-adults collected in May 2017 and readily responded with escape swims when mechanically stimulated. Post-diapausing females were individuals collected from depth (September 2016) with their first antennae (A1) folded immediately after collection, a behavioral sign of the diapause state. By 12 h post collection, all females had their A1 deployed perpendicular to the main body axis; even after deployment of the A1s, females were inactive and relatively non responsive to stimulations. These females were maintained in the laboratory during the post-diapause program.

Respiration rate experiments were run in the laboratory at the Seward Marine Center or in Fairbanks. In early May 2017, pre-adult copepodid stage CV were collected using a CalVet net (53 μm) towed vertically through the upper 100 m, individuals were live sorted under the microscope, transferred into carboys and maintained at ambient temperatures (6–7 °C). Recently molted females, which had not been mated, were used in respiration experiments on May 18 and 22 (experiment duration: 24–26 h, pre-diapause). Females in diapause were collected from depth on September 19, 2016 between 19:00 and 20:00 local time and processed as described above. Respiration experiments were conducted on the evening of September 21 (experiment duration: 5.5 h; time point: 2 days), and overnight on September 21–22 (experiment duration: 10.3 h; time point: 2–3 days) and 24–25 (experiment duration:13.3 h; time point:5 days). Incubation temperatures were between 4 and 6.5 °C. Each experiment included four blank (control) experiments containing only filtered seawater.

Oxygen consumption was measured by incubating either one (active) or one to six (post-diapause) individuals in 20 mL scintillation vials filled with freshly GFC-filtered sea water from the collection station that had been equilibrated overnight in an incubator, while being stirred gently with a magnetic stirrer to achieve uniform oxygenation. At the start of the experiment, animals were gently drawn into wide-bore pipettes and transferred to vials in the same order as subsequent measurement. A mini-stir bar was added, bubbles were purged and vials sealed with plastic scintillation-vial caps or cover slips, then submerged in the stirred water bath and placed back in the incubator. At the end of the experiment, each vial was magnetically stirred to distribute deoxygenated water evenly (checked with dye), then uncapped before the oxygen measurement. Percent saturation of O_2_ in sea water was determined with a pre-calibrated PreSens TX-3 “optode” (non-destructive optical probe: PreSens Precision Sensing GmbH, Regensburg, Germany) while measuring temperature with a ganged probe lowered to a tip-depth of 3 cm in each vial in turn. The TX-3 was set to record data every 30s and the first reading was discarded to allow settling; thereafter 3–5 readings were taken until 3 fell within 0.5% of each other and the mean of the 3 was recorded. All measurements were taken at the same temperature (±0.2 °C), as the optode was unexpectedly sensitive to temperature despite its temperature-compensating probe. The difference in percent saturation at the run temperature for each vial from the mean value of 2–4 blanks run simultaneously was converted to μg O_2_ ind^− 1^ h^− 1^ using standard tables for sea water saturation (www.EngineeringToolBox.com).

## Supplementary Information

**Additional file 1:.**

## Data Availability

The datasets for this study can be found as raw short sequences on NCBI; BioProject: PRJNA324453. The shotgun assembly used as the reference transcriptome in the article is available under the accession GFUD00000000 (PRJNA324453). The list of all differentially expressed genes (GLM), their relative expression as Log_2_ [RPKM+1] and their functional annotation is included in the Table S2. The list of DEGs from each pairwise likelihood test with their fold change expression, *P*-value and FDR are included in supplementary information files (Files S3, S4, S5, S6, S7, S8).

## Abbreviations

20E: 20-hydroxyecdysone
Genes: Shd: shade
EcR: Ecdysone receptor
USP: Ultraspiracle
Nurf-38: Nucleosome remodeling factor-38kD
cdk: Cyclin-dependent kinases
cyc: Cyclins
MAPK: Mitogen-activated protein kinases
DEGs: Differentially expressed genes
GLM: Generalized linear model
RPKM: Reads per kilobase of transcript length per million mapped reads
WGCNA: Weighted gene correlation network analysis

## References

[1] Denlinger DL (2002). Regulation of diapause. Annu Rev Entomol.

[2] Mansingh A (1971). Physiological classification of dormancies in insects. Can Entomol.

[3] Tauber CA, Tauber MJ (1981). Insect seasonal cycles: genetics and evolution. Ann Rev Ecol Evol Syst.

[4] Koštál V (2006). Eco-physiological phases of insect diapause. J Insect Physiol.

[5] Podrabsky JE, Hand SC (2015). Physiological strategies during animal diapause: lessons from brine shrimp and annual killifish. J Exp Biol.

[6] Ragland GJ, Egan SP, Feder JL, Berlocher SH, Hahn DA (2011). Developmental trajectories of gene expression reveal candidates for diapause termination: a key life-history transition in the apple maggot fly *Rhagoletis pomonella*. J Exp Biol.

[7] Hirche H-J (1996). Diapause in the marine copepod, *Calanus finmarchicus*—a review. Ophelia..

[8] Baumgartner MF, Tarrant AM (2017). The physiology and ecology of diapause in marine copepods. Annu Rev Mar Sci.

[9] Miller CB. *Neocalanus flemingeri* , a new species of Calanidae (Copepoda: Calanoida) from the subarctic Pacific Ocean, with a comparative redescription of *Neocalanus plumchrus* (Marukawa) 1921. Prog Oceanogr 1988;20:223–273, Neocalanus flemingeri, a new species of Calanidae (Copepoda: Calanoida) from the subarctic Pacific Ocean, with a comparative redescription of Neocalanus plumchrus (Marukawa) 1921, 4, DOI: 10.1016/0079-6611(88)90042-0.

[10] Miller CB, Clemons MJ (1988). Revised life history analysis for large grazing copepods in the subarctic Pacific Ocean. Prog Oceanogr.

[11] Tsuda A, Saito H, Kasai H (1999). Life histories of *Neocalanus flemingeri* and *Neocalanus plumchrus* (Calanoida: Copepoda) in the western subarctic Pacific. Mar Biol.

[12] Liu H, Hopcroft RR (2006). Growth and development of *Metridia pacifica* (Copepoda: Calanoida) in the northern Gulf of Alaska. J Plankton Res.

[13] Roncalli V, Sommer SA, Cieslak MC, Clarke C, Hopcroft RR, Lenz PH (2018). Physiological characterization of the emergence from diapause: a transcriptomics approach. Sci Rep.

[14] Roncalli V, Cieslak MC, Hopcroft RR, Lenz PH (2020). Capital breeding in a diapausing copepod: a transcriptomics analysis. Front Mar Sci.

[15] Lenz PH, Roncalli V (2019). Diapause within the context of life-history strategies in Calanid copepods (Calanoida: Crustacea). Biol Bull.

[16] Cieslak MC, Castelfranco AM, Roncalli V, Lenz PH, Hartline DK (2020). t-Distributed Stochastic Neighbor Embedding (t-SNE): A tool for eco-physiological transcriptomic analysis. Mar Genomics.

[17] Hwang DS, Han J, Won EJ, Kim DH, Jeong CB, Hwang UK, Zhou B, Choe J, Lee JS (2016). BDE-47 causes developmental retardation with down-regulated expression profiles of ecdysteroid signaling pathway-involved nuclear receptor (NR) genes in the copepod *Tigriopus japonicus*. Aquat Toxicol.

[18] Kanehisa M, Goto S (2000). KEGG: Kyoto encyclopedia of genes and genomes. Nucleic Acids Res.

[19] Jagodzik P, Tajdel-Zielinska M, Ciesla A, Marczak M, Ludwikow A (2018). Mitogen-activated protein kinase cascades in plant hormone signaling. Front Plant Sci.

[20] Lovett DL, Felder DL (1990). Ontogenetic change in digestive enzyme activity of larval and postlarval white shrimp *Penaeus setiferus* (Crustacea, Decapoda, Penaeidae). Biol Bull.

[21] Rinehart JP, Denlinger DL (2000). Heat-shock protein 90 is down-regulated during pupal diapause in the flesh fly, *Sarcophaga crassipalpis*, but remains responsive to thermal stress. Insect Mol Biol.

[22] Tammariello SP, Denlinger DL (1998). G0/G1 cell cycle arrest in the brain of *Sarcophaga crassipalpis* during pupal diapause and the expression pattern of the cell cycle regulator, proliferating cell nuclear antigen. Insect Biochem Mol Biol.

[23] Fujiwara Y, Denlinger DL (2007). High temperature and hexane break pupal diapause in the flesh fly, *Sarcophaga crassipalpis*, by activating ERK/MAPK. J Insect Physiol.

[24] Wang W, Meng B, Chen W, Ge X, Liu S, Yu J (2007). A proteomic study on postdiapaused embryonic development of brine shrimp (*Artemia franciscana*). Proteomics..

[25] Denlinger D, Yocum G, Rinehart J. Hormonal control of diapause. Insect Endocrinol; 2012. p. 430–463, Hormonal Control of Diapause, DOI: 10.1016/B978-0-12-384749-2.10010-X.

[26] Hill RJ, Billas IM, Bonneton F, Graham LD, Lawrence MC (2013). Ecdysone receptors: from the Ashburner model to structural biology. Annu Rev Entomol.

[27] Kostal V, Stetina T, Poupardin R, Korbelova J, Bruce AW (2017). Conceptual framework of the eco-physiological phases of insect diapause development justified by transcriptomic profiling. Proc Natl Acad Sci U S A.

[28] Rinehart JP, Cikra-Ireland RA, Flannagan RD, Denlinger DL (2001). Expression of ecdysone receptor is unaffected by pupal diapause in the flesh fly, *Sarcophaga crassipalpis*, while its dimerization partner, USP, is downregulated. J Insect Physiol.

[29] Ragland GJ, Fuller J, Feder JL, Hahn DA (2009). Biphasic metabolic rate trajectory of pupal diapause termination and post-diapause development in a tephritid fly. J Insect Physiol.

[30] Ingvarsdottir A, Houlihan DF, Heath MR, Hay SJ (1999). Seasonal changes in respiration rates of *Calanus finmarchicus* (Gunnerus) at copepodite stage V. Fish Oceanogr.

[31] Saumweber WJ, Durbin EG (2006). Estimating potential diapause duration in *Calanus finmarchicus*. Deep-Sea Res II Top Stud Oceanogr.

[32] Beisel C, Paro R (2011). Silencing chromatin: comparing modes and mechanisms. Nat Rev Genet.

[33] Lenz PH, Roncalli V, Cieslak MC, Tarrant AM, Castelfranco AM, Hartline DK. Diapause vs. reproductive programs: transcriptional phenotypes in a keystone copepod. Commun Biol. 2021;4(1):1–13. 10.1038/s42003-021-01946-0 PMC8007741 33782539

[34] Whitfield W, Gonzalez C, Maldonado-Codina G, Glover DM (1990). The A-and B-type cyclins of Drosophila are accumulated and destroyed in temporally distinct events that define separable phases of the G2-M transition. EMBO J.

[35] Monell, K. Characterization of cell division in the tissues of the calanoid copepod *Neocalanus flemingeri* from diapause through early oogenesis. MS Thesis University of Hawai’i at Manoa. http://hdl.handle.net/10125/73356.

[36] Roncalli V, Cieslak MC, Sommer SA, Hopcroft RR, Lenz PH (2018). *De novo* transcriptome assembly of the calanoid copepod *Neocalanus flemingeri*: a new resource for emergence from diapause. Mar Genomics.

[37] Langmead B, Trapnell C, Pop M, Salzberg SL (2009). Ultrafast and memory-efficient alignment of short DNA sequences to the human genome. Genome Biol.

[38] Bray NL, Pimentel H, Melsted P, Pachter L (2016). Near-optimal probabilistic RNA-seq quantification. Nat Biotechnol.

[39] Mortazavi A, Williams BA, McCue K, Schaeffer L, Wold B (2008). Mapping and quantifying mammalian transcriptomes by RNA-Seq. Nat Methods.

[40] van der Maaten L, Hinton G (2008). Visualizing data using t-SNE. J Mach Learn Res.

[41] Krijthe JH. Rtsne: T-distributed stochastic neighbor embedding using Barnes-Hut implementation. R package version 013, URL https://github.com/jkrijthe/Rtsne. 2015.

[42] Ester M, Kriegel H-P, Sander J, Xu X, editors. A density-based algorithm for discovering clusters in large spatial databases with noise. Kdd; 1996.

[43] Dunn JC (1974). Well-separated clusters and optimal fuzzy partitions. J Cybernetics.

[44] Hahsler M, Piekenbrock M, Arya S, Mount D. dbscan: Density Based Clustering of Applications with Noise (DBSCAN) and related algorithms. R package version. 2017:1.0.

[45] Desgraupes B. clusterCrit: Clustering Indices. R package version 1.2. 6. 2015.

[46] Schaeffer L, Pimentel H, Bray N, Melsted P, Pachter L. Pseudoalignment for metagenomic read assignment. Bioinformatics. 2017;33(14):2082–8. 10.1093/bioinformatics/btx106. 10.1093/bioinformatics/btx106 PMC5870846 28334086

[47] Larsson J. Eulerr: area-proportional Euler and Venn diagrams with ellipses. R package version. 2018;4(0).

[48] Langfelder P, Horvath S (2008). WGCNA: an R package for weighted correlation network analysis. BMC Bioinform.

[49] Alexa A, Rahnenfuhrer J. Gene set enrichment analysis with topGO. Bioconductor Improv. 2009;27:1–26.

