# Correction to: Post-diapause transcriptomic restarts: insight from a high-latitude copepod

**DOI:** 10.1186/s12864-021-07868-9

**Published:** 2021-09-14

**Authors:** Vittoria Roncalli, Matthew C. Cieslak, Ann M. Castelfranco, Russell R. Hopcroft, Daniel K. Hartline, Petra H. Lenz

**Affiliations:** 1 Pacific Biosciences Research Center, University of Hawai’i at Mānoa, 1993 East-West Rd, Honolulu, HI 96822 USA; 2 Integrative Marine Ecology Department, Stazione Zoologica Anton Dohrn, Naples, Italy; 3 Institute of Marine Science, University of Alaska, Fairbanks, 120 O’Neill, Fairbanks, AK 99775-7220 USA

**Correction to: BMC Genomics 22, 409 (2021)**

**https://doi.org/10.1186/s12864-021-07557-7**

Following publication of the original article [1], it was reported that due to a typesetting error the incorrect versions of Figs. 4, 7, 9 and 10 were published. The correct figures are given in this Correction article.

**Fig. 4:**
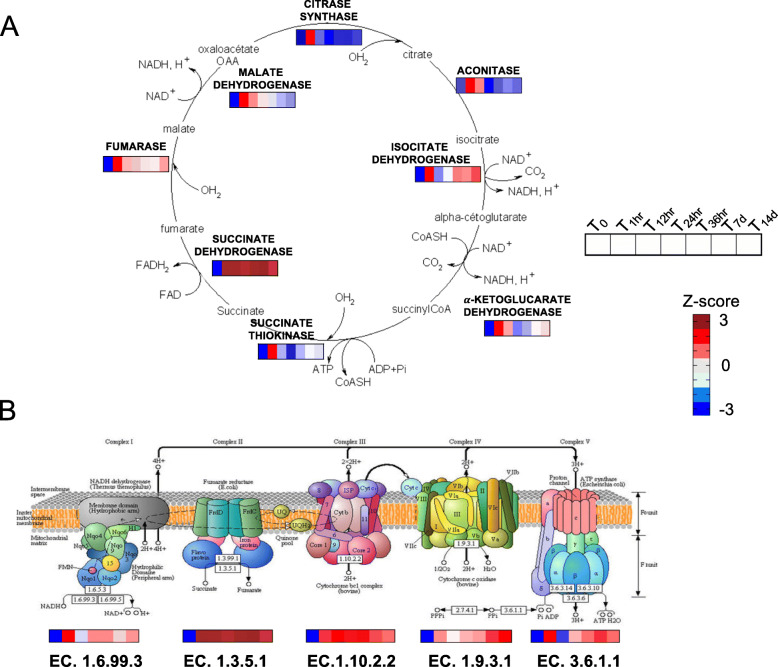
Tricarboxylic acid cycle (TCA) and oxidative phosphorylation. **a** Schematic representation for tricarboxylic acid cycle (TCA) adapted from Wikimedia Commons (https://commons.wikimedia.org/wiki/File:Cycle_de_krebs.png). For each step of the TCA cycle intermediate products, enzymes (bold) and coenzymes (FAD and NAD+) are indicated. For each enzyme, heatmaps show relative expression (z-score) in females from T0 to T14d. **b** KEGG pathway diagram (map 00190) including gene expression results for the five genes among the DEGs in *N. flemingeri*. The upper part of the figure shows the five respiratory chain complexes with the corresponding E.C. numbers for each enzyme. In the bottom part, heatmaps show relative expression (z-score) of each enzyme associated with the respiratory chain complex in females from T0 to T14d. All enzymes shown were identified as DEGs. Copyright permission to use and adapt the KEGG map 00190 has been granted from KEGG database [18]

**Fig. 7:**
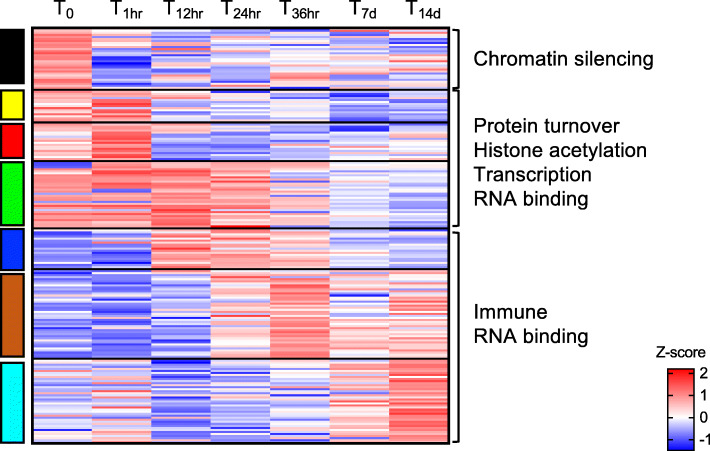
RNA metabolism and chromatin silencing. Heatmap of the differentially expressed genes (*n* = 198) annotated with GO terms associated with chromatin silencing and RNA metabolism (see Fig. 6). Genes (rows) were ordered based on modules (left) for which they were enriched (see Fig. 5). For each gene, relative expression is shown as the average z-score for each timepoint as indicated by the color scale. Timepoints are indicated at the top of the heatmap. Labels on the right indicate processes that were highly represented in each module

**Fig. 9:**
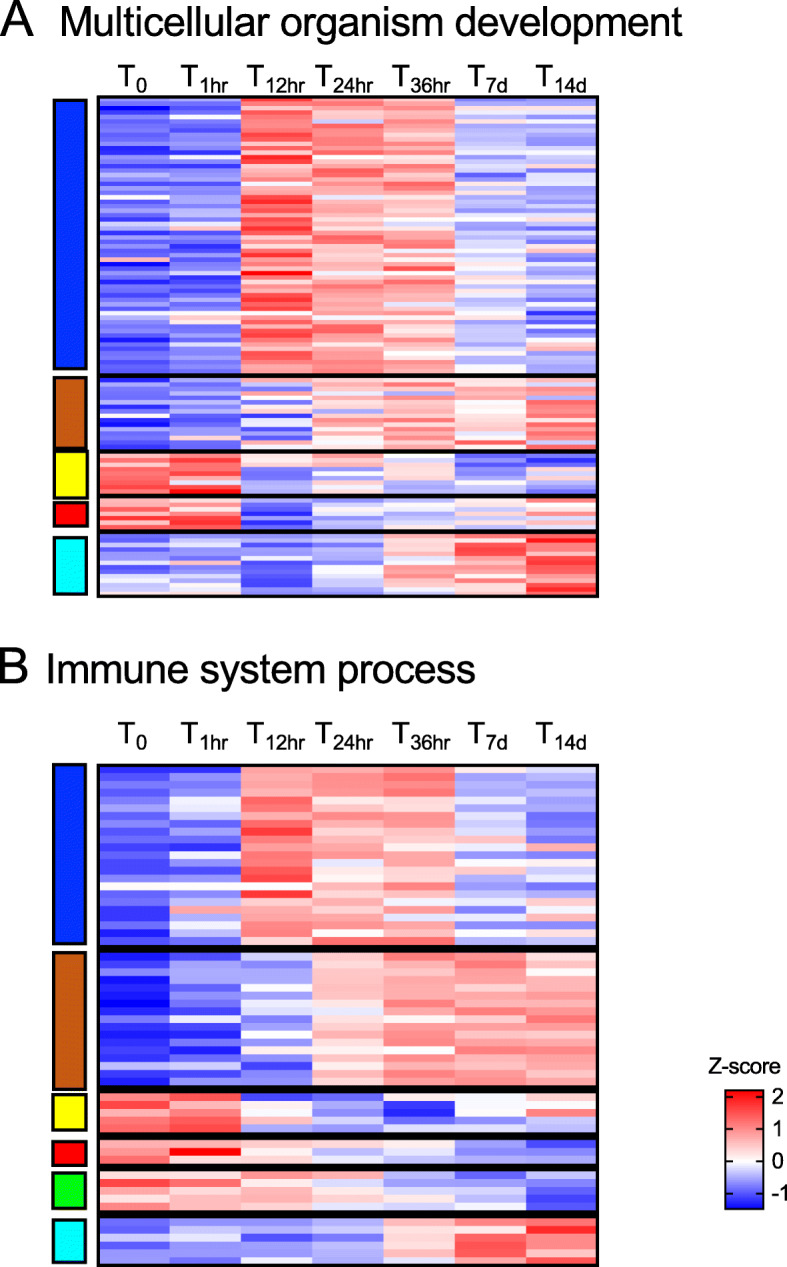
Multicellular organism development and immune system process. Heatmap of the differentially expressed genes annotated with GO terms associated with: **a** multicellular organism development (*n* = 108) and **b** immune system process (*n* = 61) (see Fig. 6). Genes (rows) were ordered based on modules (left) for which they were enriched (see Fig. 5). For each gene, relative expression is shown as the average z-score for each timepoint as indicated by the color scale. Timepoints are indicated at the top of the heatmap

**Fig. 10:**
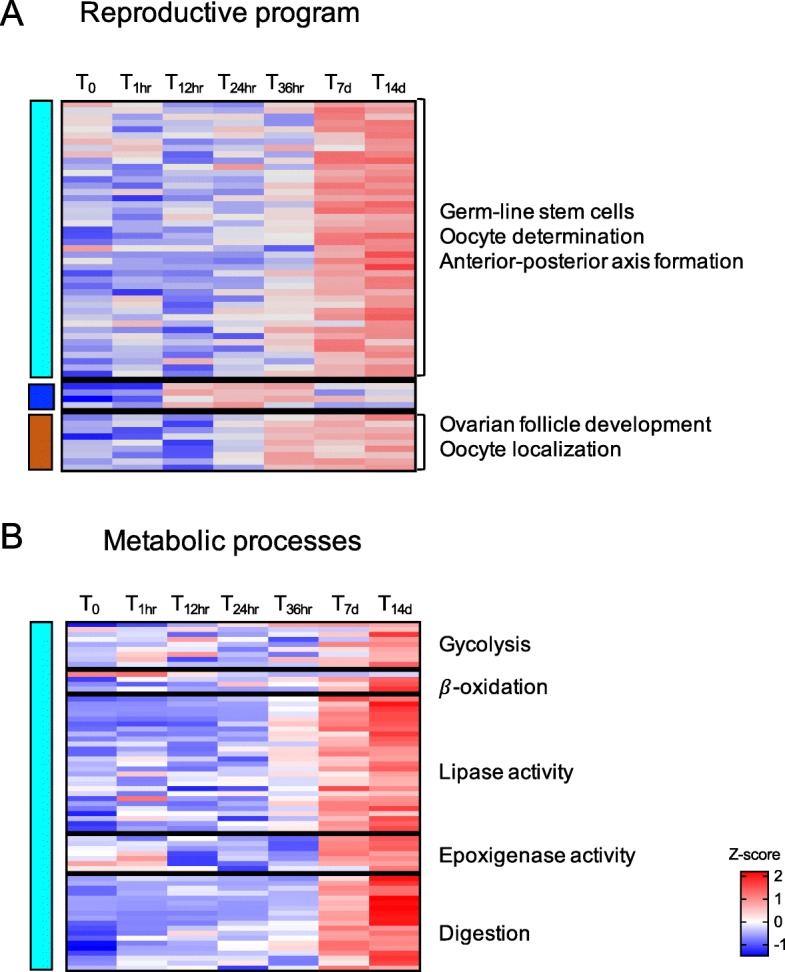
Reproductive program and metabolic processess. Heatmap of the differentially expressed genes annotated with GO terms associated with **a** oogenesis (*n* = 54) and **b** metabolic processes: glycolysis (*n* = 9), β-oxidation (*n* = 4), lipase activity (*n* = 27), epoxigenase activity (*n* = 9) and digestion (*n* = 19) (see Fig. 6). Genes (rows) were ordered based on modules (left) for which they were enriched (see Fig. 5). For each gene, relative expression is shown as the average z-score for each timepoint as indicated by the color scale. Timepoints are indicated at the top of the heatmap. Labels on the right indicate processes that were highly represented in each module

The original article has been updated.

## References

[1] Roncalli V, Cieslak MC, Castelfranco AM, Hopcroft RR, Hartline DK, Lenz PH (2021). Post-diapause transcriptomic restarts: insight from a high-latitude copepod. BMC Genomics.

[43] Desgraupes B (2015). clusterCrit: Clustering Indices. R package version 1.2. 6.

