# Supplementary material for: Post-diapause transcriptomic restarts: insight from a high-latitude copepod
Source: BMC Genomics. 2021 Jun 3;22:409. doi: 10.1186/s12864-021-07557-7 (PMC8176732; doi:10.1186/s12864-021-07557-7)
Supplement: Supplementary file 1 — Additional file 1:. [file 12864_2021_7557_MOESM1_ESM.zip › Roncalli-Supplementary-FINALdocx.docx]

**Supplementary**

**Table S1.** **Summary of differential gene expression analysis from T_0_ to T_14d_**. Differentially expressed genes were identified using a general linear model (GLM) followed by likelihood ratio tests (FDR; p-value ≤ 0.05) between females collected at sequential time points. For each pairwise likelihood test, the number of total DEGs, up- and down-regulated is listed.

|  | **DEGs** | | |
| --- | --- | --- | --- |
|  | Total | Up-regulated | Down-regulated |
| Generalized linear model (GLM) | 14608 |  |  |
| Likelihood ratio tests |  |  |  |
| T_0_ vs T_1hr_ | 883 | 502 | 381 |
| T_1hr_ vs T_12hr_ | 1443 | 941 | 502 |
| T_12hr_ vs T_24hr_ | 830 | 365 | 465 |
| T_24hr_ vs T_36hr_ | 693 | 334 | 359 |
| T_36hr_ vs T_7d_ | 770 | 474 | 296 |
| T_7d_ vs T_14d_ | 790 | 461 | 329 |

**Table S2.** **Relative expression and functional annotation of the differentially expressed genes identified in females from T_0_ to T_14d_.** List of all DEGs identified using the general linear model (GLM). Relative expression is provided as RPKM (Log_2_[RPKM+1]). Genes were annotated against SwissProt and Gene Ontology databases (n=5,348) (see text). For each gene, annotation name, E value, UniprotID, GO description and GO ID and enzyme commission (EC) number are listed.

**Figure S1.**  **Two commonly-used dimensionality-reduction algorithms applied to the same data set~~s~~ as t-SNE.**  The log-transformed relative expression levels (Log_2_(RPKM+1)) of all genes (n=140,841) were used in both algorithms for comparison with Figure 1 in the main text. A) Principal component analysis (1,2)(PCA; first two components), with four clusters identified by DBSCAN (see Methods in main text). Cluster 1 contains all but one of the earliest samples (T_0_ and T_1hr_), while 2 and 3 contain, on average, progressively later samples, albeit the separation is not perfect. Cluster 4 contains a mix of three samples from early and late times. B) Hierarchical clustering using the one-dimensional *hclust* algorithm (3) produces three clusters of samples. It separates all of the earliest time points (T_0_ and T_1hr_) into a single cluster as does t-SNE (open symbols). Clusters 2 and 3 correspond approximately to those in the PCA plot (A). The trend in temporal progression is shown in the bar diagram below. The two algorithms are in general agreement with the clustering by t-SNE (Fig. 1, main text) but the separation between the diapause and post-diapause phenotypes is less pronounced. The t-SNE plot thus provides a clearer visualization of the transition from diapause to post-diapause phenotypes.

**File S3.** **List of differentially expressed genes identified in females from T_0_ to T_1hr_.** The DEGs were identified using the general linear model (GLM) followed by pairwise comparison between T_0_ and T_1hr_ females using an FDR adjusted P-value of < 5%. As implemented by edgeR the file reports, for each gene, fold change expression (Log_2_), expression level as logarithm of counts per million reads (LogCPM), P-values and adjusted P-values for false discovery rate (FDR).

**File S4.** **List of differentially expressed genes identified in females from T_0_ to T_12hr_.** The DEGs were identified using the general linear model (GLM) followed by pairwise comparison between T_0_ and T_12hr_ females using an FDR adjusted P-value of < 5%. As implemented by edgeR the file reports, for each gene, fold change expression (Log_2_), expression level as logarithm of counts per million reads (LogCPM), P-values and adjusted P-values for false discovery rate (FDR).

**File S5.** **List of differentially expressed genes identified in females from T_0_ to T_24hr_.** The DEGs were identified using the general linear model (GLM) followed by pairwise comparison between T_0_ and T_24hr_ females using an FDR adjusted P-value of < 5%. As implemented by edgeR the file reports, for each gene, fold change expression (Log_2_), expression level as logarithm of counts per million reads (LogCPM), P-values and adjusted P-values for false discovery rate (FDR).

**File S6.** **List of differentially expressed genes identified in females from T_0_ to T_36hr_.** The DEGs were identified using the general linear model (GLM) followed by pairwise comparison between T_0_ and T_36hr_ females using an FDR adjusted P-value of < 5%. As implemented by edgeR the file reports, for each gene, fold change expression (Log_2_), expression level as logarithm of counts per million reads (LogCPM), P-values and adjusted P-values for false discovery rate (FDR).

**File S7.** **List of differentially expressed genes identified in females from T_0_ to T_7d_.** The DEGs were identified using the general linear model (GLM) followed by pairwise comparison between T_0_ and T_7d_ females using an FDR adjusted P-value of < 5%. As implemented by edgeR the file reports, for each gene, fold change expression (Log_2_), expression level as logarithm of counts per million reads (LogCPM), P-values and adjusted P-values for false discovery rate (FDR).

**File S8.** **List of differentially expressed genes identified in females from T_0_ to T_14d_.** The DEGs were identified using the general linear model (GLM) followed by pairwise comparison between T_0_ and T_14d_ females using an FDR adjusted P-value of < 5%. As implemented by edgeR the file reports, for each gene, fold change expression (Log_2_), expression level as logarithm of counts per million reads (LogCPM), P-values and adjusted P-values for false discovery rate (FDR).

**References**

1. Hotelling H. Analysis of a complex of statistical variables into principal components. J. Educ. Psychol. 1933;24, 417.
2. Hartigan JA. Clustering algorithms. John Wiley & Sons, Inc.;1975.
3. Gordon A. Classification (2nd edition) Chapman and Hall/CRC Press,
   London;1999.
