# Supplementary figures and images for: Post-diapause transcriptomic restarts: insight from a high-latitude copepod
Source: BMC Genomics. 2021 Jun 3;22:409. doi: 10.1186/s12864-021-07557-7 (PMC8176732; doi:10.1186/s12864-021-07557-7)

## A. PCA

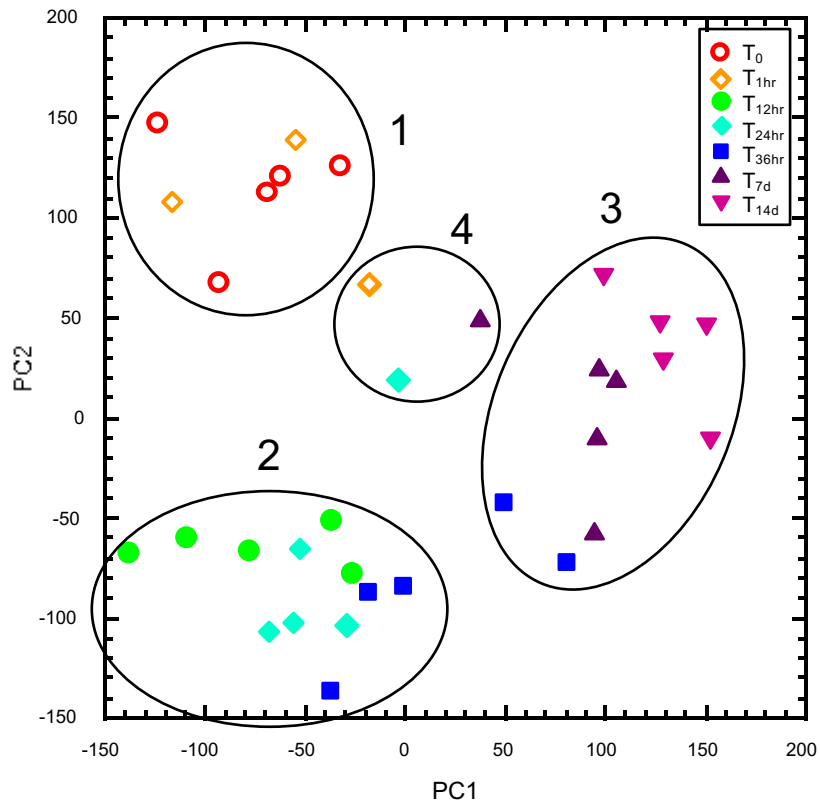

## B. hclust

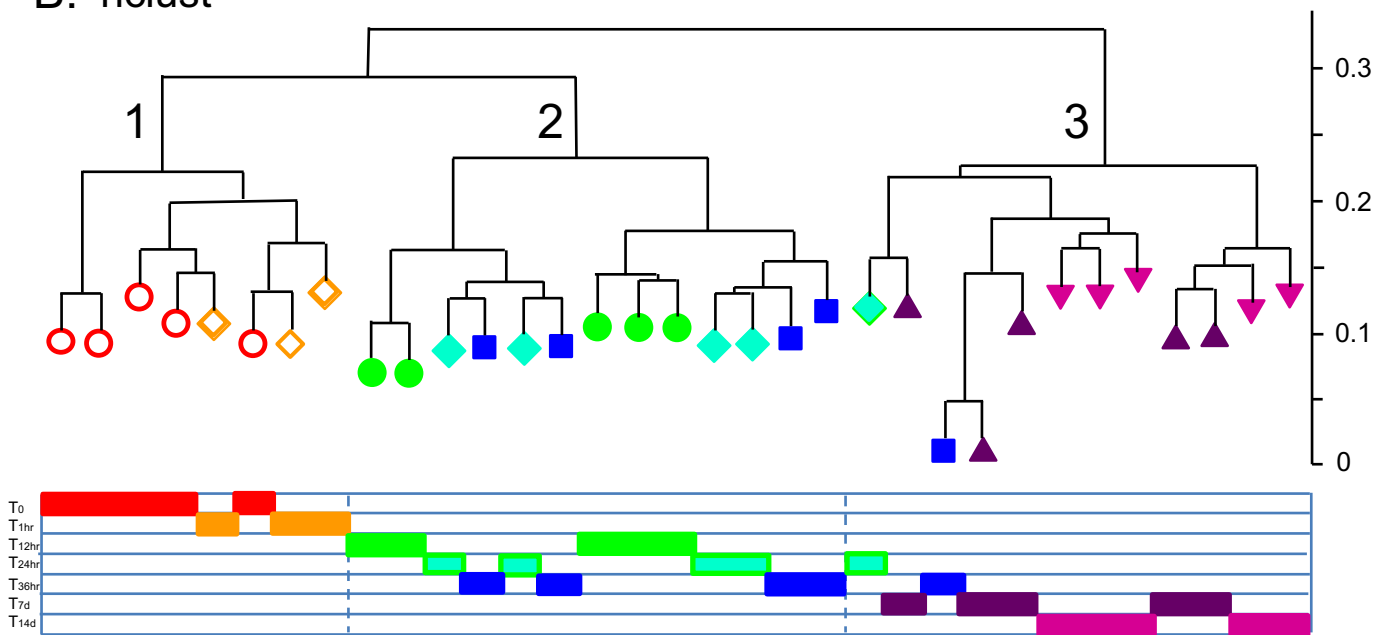

Supplement: Supplementary file 1 — Additional file 1:. [file 12864_2021_7557_MOESM1_ESM.zip › FigS1.pdf]
